# Adherence to Prescribed E-Diary Recording by Patients With Seasonal Allergic Rhinitis: Observational Study

**DOI:** 10.2196/16642

**Published:** 2020-03-16

**Authors:** Marco Di Fraia, Salvatore Tripodi, Stefania Arasi, Stephanie Dramburg, Sveva Castelli, Danilo Villalta, Francesca Buzzulini, Ifigenia Sfika, Valeria Villella, Ekaterina Potapova, Serena Perna, Maria Antonia Brighetti, Alessandro Travaglini, Pierluigi Verardo, Simone Pelosi, Anna Maria Zicari, Paolo Maria Matricardi

**Affiliations:** 1 Department of Pediatric Pulmonology, Immunology and Intensive Care Medicine Charité University Medicine Berlin Germany; 2 Department of Pediatrics Sapienza University of Rome Rome Italy; 3 Pediatric Allergology Unit Sandro Pertini Hospital Rome Italy; 4 Allergology Service Policlinico Casilino Rome Italy; 5 TPS Production Rome Italy; 6 Pediatric Allergology Unit Department of Pediatric Medicine Bambino Gesù Children's Research Hospital Rome Italy; 7 Department of Immunology-Allergy Santa Maria degli Angeli Hospital Pordenone Italy; 8 Department of Biology University of Rome Tor Vergata Rome Italy; 9 Center of Aerobiology Agenzia Regionale per la Protezione Ambientale Pordenone Italy

**Keywords:** mobile health, e-Diary, precision medicine, pollen, seasonal allergic rhinitis, blended care

## Abstract

**Background:**

Complete diagnosis and therapy of seasonal allergic rhinoconjunctivitis require evidence that exposure to the sensitizing pollen triggers allergic symptoms. Electronic clinical diaries, by recording disease severity scores and pollen exposure, can demonstrate this association. However, patients who spontaneously download an e-diary app show very low adherence to their recording.

**Objective:**

The objective of our study was to assess adherence of patients with seasonal allergic rhinitis to symptom recording via e-diary explicitly prescribed by an allergist within a blended care approach.

**Methods:**

The @IT-2020 project is investigating the diagnostic synergy of mobile health and molecular allergology in patients with seasonal allergic rhinitis. In the pilot phase of the study, we recruited Italian children (Rome, Italy) and adults (Pordenone, Italy) with seasonal allergic rhinitis and instructed them to record their symptoms, medication intake, and general conditions daily through a mobile app (Allergy.Monitor) during the relevant pollen season.

**Results:**

Overall, we recruited 101 Italian children (Rome) and 93 adults (Pordenone) with seasonal allergic rhinitis. Adherence to device use slowly declined during monitoring in 3 phases: phase A: first week, ≥1267/1358, 90%; phase B: second to sixth week, 4992/5884, 80% to 90%; and phase C: seventh week onward, 2063/2606, 70% to 80%. At the individual level, the adherence assessed in the second and third weeks of recording predicted with enough confidence (Rome: Spearman ρ=0.75; *P*<.001; Pordenone: ρ=0.81; *P*<.001) the overall patient adherence to recording and was inversely related to postponed reporting (ρ=–0.55; *P*<.001; in both centers). Recording adherence was significantly higher during the peak grass pollen season in Rome, but not in Pordenone.

**Conclusions:**

Adherence to daily recording in an e-diary, prescribed and motivated by an allergist in a blended care setting, was very high. This observation supports the use of e-diaries in addition to face-to-face visits for diagnosis and treatment of seasonal allergic rhinitis and deserves further investigation in real-life contexts.

## Introduction

### Background

Seasonal allergic rhinoconjunctivitis (SAR) affects patients exposed to pollens to which they are sensitized. The etiological diagnosis and therapy of SAR require a demonstration that exposure to the sensitizing pollen triggers allergic symptoms [1]. Objectively, this link is established by a positive outcome to nasal allergen provocation tests [2] or allergen exposure in pollen chambers [3]. Unfortunately, both these tests are costly and time consuming and are mostly used in clinical trials [4]. In clinical daily life, a causality between pollen exposure and symptoms is often assessed by a careful *retrospective* clinical history [5]. However, recall biases make the diagnosis based on retrospective data somewhat imprecise, especially in patients apparently sensitized to multiple pollens that share the same pollination periods [6], which is a frequent setting in Mediterranean countries [7].

This diagnostic problem can be partially solved through a *prospective* clinical history, based on the patient’s daily recording of symptoms and medication intake in a clinical diary [8]. Indeed, the trajectories of daily symptom scores or a combined symptom and medication score (CSMS) are free from recall bias and can be matched with daily concentration counts, obtained in parallel, of the potentially eliciting pollen sources [9,10]. While traditional and time-consuming clinical diaries on paper records are rarely used, electronic clinical diaries (e-diaries) have become increasingly prevalent [11,12]. E-diaries are apps consisting of short questionnaires filled in daily by the patient, usually on his or her mobile phone or tablet computer [11-13]. Recording e-diaries is easy and quick, and the software automatically provides daily scores, time trajectories, and descriptive reports [8,10-16].

Several e-diaries are available for pollen allergies in European countries, and some of them have also been used in trials or observational studies [8,10,12,14-19]. In most of the study settings, the app was directly downloaded by the patients, with no or only occasional intervention by their allergist [14-17]. The observational studies were characterized by large population size (more than 9000 participants) and big datasets (112,054 registered visual analog scale [VAS] data) [14], balanced by a poor mean adherence (<10%) to daily recording [14,15].

### Objective

We hypothesized that the patients’ adherence to recording of e-diaries would be significantly increased if the rationale and the use of the e-diary were personally explained by an allergist to the patient (blended approach). To test this hypothesis, we examined the rate and cofactors of adherence to recording of an e-diary among Italian patients with SAR participating in the @IT-2020 project, a study of combined molecular diagnostics and mobile health for allergen immunotherapy in patients with SAR.

## Methods

### @IT-2020 Project

The pilot study of the @IT-2020 project was carried out in 2 Italian centers differing significantly in terms of environmental setting and patient characteristics.

### Climate and Study Area

Pordenone, Italy, is a city with about 50,000 inhabitants, which extends over an area of 38 km^2^ [20]. Pordenone is 600 km north of Rome and the territory is located in northeastern Italy, about 50 km from the Adriatic Sea, in the Po-Veneto plain south of the Carnic Pre-Alps, in the continental biogeographical region [21]. It has a mean annual temperature of 13.1°C and mean rainfall of 1292 mm [22].

Rome, Italy, has 3 million inhabitants in an area of almost 1300 km^2^ [20] and is 20 km from the Tyrrhenian Sea. Rome is located in the Mediterranean biogeographical region [21] with mean annual temperature of 15.7°C and mean rainfall of 798 mm (Rome Monte Mario) [22].

### Study Population

Between November 2016 and February 2017, we recruited 101 children aged 10 to 18 years at Ospedale Sandro Pertini in Rome and 93 adults aged over 18 years at Ospedale Santa Maria degli Angeli in Pordenone. Criteria for eligibility were (1) being followed up for at least one year for allergic rhinoconjunctivitis (objectively confirmed by skin prick tests or in vitro immunoglobulin E tests, or both) due to outdoor aeroallergens (pollen or spores), (2) residing within 30 km of the aerobiological station of the study center, (3) having no intention to change residence in the 6 months after recruitment, and (4) being able to use a mobile phone (by the patient or the patient’s parents). Exclusion criteria were (1) previous allergen immunotherapy for any outdoor allergen, and (2) any other severe nonatopic chronic disease. All participants (in the case of children, their parents or guardians) provided informed written consent to the clinical investigations.

### Study Design

Recruited patients underwent a first clinical assessment (T0), including clinical questionnaires, during which they were instructed on the use of the Allergy.Monitor (Technology Project and Software [TPS] Production, Rome, Italy) mobile app to monitor their symptoms and medication intake during the following study period. According to the timing of retrospective symptoms and skin prick test results, participants were assigned an individual monitoring period during the suspected high season of the putative eliciting pollen. During this period, participants were asked to monitor their eye, nose, and lung symptoms, as well as their effect on daily activities and daily medication intake, and report them via Allergy.Monitor. After the monitoring period, all participants underwent a second clinical assessment (T1), including a repetition of the initial clinical questionnaires focused on the past pollen season, internationally validated by the International Study of Asthma and Allergies in Childhood [23], the Allergic Rhinitis and its Impact on Asthma (ARIA) initiative [24,25], and the Global Initiative for Asthma [26]. The study design and procedures had been approved by the ethics committee of each participating center.

### Skin Prick Tests

**S**kin prick tests were performed using a standard panel of commercial extracts (ALK-Abelló, Milan, Italy) of outdoor and indoor aeroallergens (*Alternaria*, Bermuda grass, birch, cat dander, cypress, dog dander, hazel, house dust mite, mugwort, olive tree, plane tree, ragweed, Russian thistle, timothy grass, and pellitory-of-the-wall). Histamine 0.1 mg/mL and glycerol solution were used as positive and negative controls, respectively. Morrow Brown needles were used to prick the skin and the wheal reactions were read after 15 minutes. A wheal equal to or greater than 3 mm after subtraction of the negative control was regarded as positive.

### Pollen Counts and Pollen Periods

The pollen count data, acquired from March 1 to September 30, 2016, were provided by the pollen stations of Rome (Tor Vergata University) and Pordenone (Agenzia Regionale per la Protezione dell’Ambiente del Friuli Venezia Giulia). Pollen was collected using a VPPS 2000 pollen sampler (Lanzoni srl, Bologna, Italy), and data were acquired as reported in Standard UNI CEN/TS 16868:2015 [27]. Pollen periods were determined (1) according to the 2017 European Academy of Allergy and Clinical Immunology (EAACI) position paper on pollen exposure times [28] (EAACI criteria), and (2) by adapting these criteria to the pollen situation in Italy (local criteria).

### Allergy.Monitor

**Figure 1:**
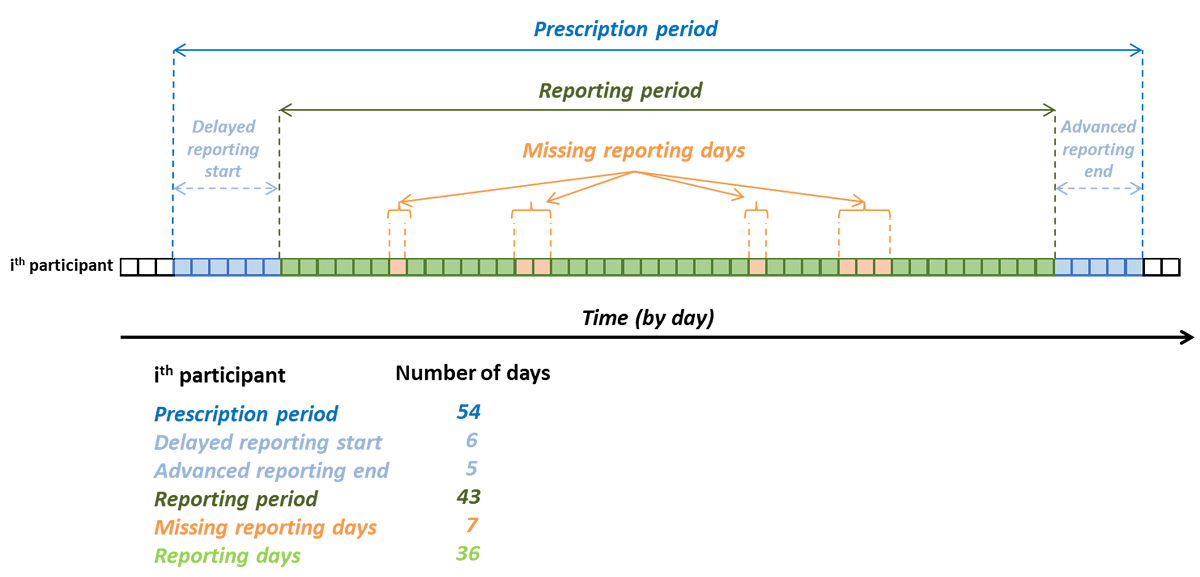
Graphical representation of definitions used in this study. The box line represents the monitored period (each box is a specific day) of a hypothetical participant. In this example the medical doctor, according to the individual participant’s clinical history, invited the patient to fill in the e-diary questionnaire for 54 days (prescription period). The patient started to record symptoms 6 days after the prescribed beginning day (delayed reporting start) and finished recording symptoms 5 days before the prescribed ending day (advanced reporting end). Thus, the reporting period lasted 43 days, during which the participant did not fill in the e-diary questionnaire for 7 days (missing reporting days). Overall, the participant filled in the e-diary questionnaire for 36 days (reporting days).

Allergy.Monitor is a mobile app designed for daily reporting of symptoms and medication intake related to allergic rhinitis or asthma. In this study, medical doctors, on the basis of clinical history, defined a time frame (*prescription period*; Figure 1) for each patient, in which he or she was encouraged to fill in a daily questionnaire regarding his or her symptoms and medication intake. The system offers a bidirectional interaction between physician and patient via email, chat, and text messaging. Patients not entering their data for 2 consecutive days received an automatic alert message on their mobile phone or by email; after 4 days without reporting, the alert was followed by a phone call from the physician or nurse. The patient could insert data referring to 1 day only on the same day or on the following one (*postponed reporting*). For each participant, *adherence to prescription* was calculated as the number of actual reporting days / prescription period ×100; *adherence* was calculated as the number of actual reporting days / reporting period ×100; and postponed reporting was calculated as the number of postponed reporting days / actual reporting days ×100.

### Symptom and Medication Scores

We used the following symptom and medication scores in this study: Rhinoconjunctivitis Total Symptom Score (RTSS; score 0-18) [29]; CSMS (score 0-6) [30]; and VAS (score 0-10) [31]. RTSS and CSMS were calculated automatically by the Allergy.Monitor app, for every reporting day, on the basis of 4 questions on nasal symptoms (sneezing, rhinorrhea, nasal pruritus, nasal congestion), 2 on ocular symptoms (itchy eyes, watery eyes), and 3 questions on medication intake (antihistaminic drugs, local corticosteroids, systemic corticosteroids). The severity of each of the symptoms was also measured by the patient using 4 different emoticons, each representing a distinct severity grade (no symptoms, mild, moderate, or severe). Overall severity was also measured by a VAS in response to the question “How do you feel in relation to your allergic symptoms today?”

### Statistics

We summarized data as numbers (n) and frequencies (%) if they were categorical and as mean or median and standard deviation or interquartile range if quantitative. We examined all described analyses for each of the study centers (Rome and Pordenone). We evaluated the prevalence of atopic sensitization (skin prick test ≥3 mm) to airborne allergens. For every pollen period considered, we calculated adherence values (see above for definition) for each participant and compared their means using a nonparametric Friedman test for repeated measures. We adjusted the *P* of multiple comparison by the Bonferroni correction. We studied adherence trends over time considering the time (in days) that had passed since the first day of the reporting period. We used the Spearman rank correlation coefficient to investigate the relationship between total adherence (%), postponed reporting (%), and adherence achieved between the seventh and the 21st reporting day (%). Mean CSMS scores by time were computed for the local whole season. We considered *P*<.05 to be statistically significant. Statistical analyses were performed with R version 3.2.3 (R Foundation).

## Results

### Study Population

Overall, 101 children (Rome) and 93 adults (Pordenone) with mean (SD) ages of 13.7 (SD 2.8) and 34.3 (14.4), respectively, met the inclusion criteria (Table 1). Male sex was slightly more frequent in both populations: 62.4% (63/101) for Rome and 56% (52/93) for Pordenone. At T0, according to the ARIA questionnaire, the population in Pordenone was characterized by a higher prevalence of moderate to severe (intermittent and persistent) allergic rhinitis than in Rome (90/93, 97% vs 51/101, 50.5%, respectively). At T1, this difference was less evident (64/75, 85% vs 68/91, 75%). The prevalence of allergic asthma was similar in both groups (Rome: 28/101, 27.7%; Pordenone: 24/93, 26%), whereas the Rome population seemed to be more affected by oral allergy syndrome, urticaria, atopic dermatitis, and anaphylaxis (Table 1). Grass pollen was the most relevant allergen in both study populations. Positive skin prick test reactions to olive tree and cypress were more frequent in Rome, while sensitization to birch was more prevalent in Pordenone. Sensitization to indoor allergens was equally prevalent in both populations.

**Table 1:** Characteristics of the study population.

Characteristic				Rome (n=101)		Pordenone (n=93)
Males, n (%)				63 (62.4)		52 (56)
Age (years), mean (SD)				13.7 (2.8)		34.3 (14)
**Allergic rhinitis, n (%)**						
	Age at onset (years), median (IQR^a^)			6 (4-8)		15 (8-22)
	**ARIA^b^ classification at T0**					
		Mild intermittent	19 (18.8)		1 (1)	
		Mild persistent	31 (30.7)		2 (2)	
		Moderate to severe intermittent	11 (10.9)		17 (18)	
		Moderate to severe persistent	40 (39.6)		73 (79)	
	**ARIA classification at T1^c^, n (%)**					
		Mild intermittent	6 (7)		2 (3)	
		Mild persistent	17 (19)		9 (12)	
		Moderate to severe intermittent	4 (4)		13 (17)	
		Moderate to severe persistent	64 (70)		51 (68)	
**Other allergic comorbidities, n (%)**						
	Allergic asthma		28 (27.7)		24 (26)	
	Oral allergic syndrome		32 (32.3)		23 (25)	
	Urticaria or angioedema		19 (19.2)		8 (9)	
	Atopic dermatitis		28 (28.3)		11 (12)	
	Gastrointestinal disorders		4 (4.0)		1 (1)	
	Anaphylaxis episode		10 (10.1)		1 (1)	
	Other		5 (5.1)		2 (2)	

^a^IQR: interquartile range.

^b^ARIA: Allergic Rhinitis and its Impact on Asthma.

^c^Study population at T1: Rome, n=91; Pordenone, n=75.

### Pollen Periods

The graphical representation of grass pollen counts (grains/m^3^) highlighted differences between the 2 cities. The maximum grass pollen count in Rome (199 grains/m^3^) was higher than in Pordenone (145 grains/m^3^), and the grass pollination period was longer in Rome. Grass pollen periods in 2016 differed significantly if calculated according to EAACI criteria or local criteria (Table 2). While we used EAACI criteria for their reproducibility and standardization, the application of locally adapted criteria resulted in shorter and less fragmented periods.

**Table 2:** Grass pollen period criteria and duration, by study center.

Pollen period	Criteria	Rome (n=101)			Pordenone (n=93)		
		No. of time intervals^a^	Cumulative duration (days), n	Adherence mean (95% CI) (%)	No. of time intervals^a^	Cumulative duration (days), n	Adherence mean (95% CI) (%)
EAACI whole season^b^	5 days (out of 7 consecutive days) each with ≥3 pollen grains/m^3^ and with a sum of ≥30 pollen grains/m^3^	2	132	82.1 (79.3-84.9)	2	132	86.3 (83.5-89.2)
Local whole season^c^	5 days (out of 7 consecutive days) each with ≥10 pollen grains/m^3^ and with a sum of ≥100 pollen grains/m^3^	1	97	81.9 (79.1-84.8)	2	72	86.2 (83.1-89.2)
EAACI peak season^b^	3 consecutive days, each with ≥50 pollen grains/m^3^	5	28	81.0 (77.6-84.4)	2	13	90.4 (87.5-93.3)
Local peak season^c^	3 days (out of 5 consecutive days) each with ≥50 pollen grains/m^3^	1	55	81.0 (78.0-84.1)	2	24	90.3 (87.6-92.9)
EAACI high days^b^	Days with at least 50 pollen grains/m^3^	18	45	80.9 (77.9-84.0)	9	23	89.3 (86.4-92.1)

^a^See Figure 1 for specifications of time periods.

^b^European Academy of Allergy and Clinical Immunology (EAACI) criteria [28].

^c^Adaptation of EAACI criteria to the local scenario.

### Adherence to E-Diary Recording

**Figure 2:**
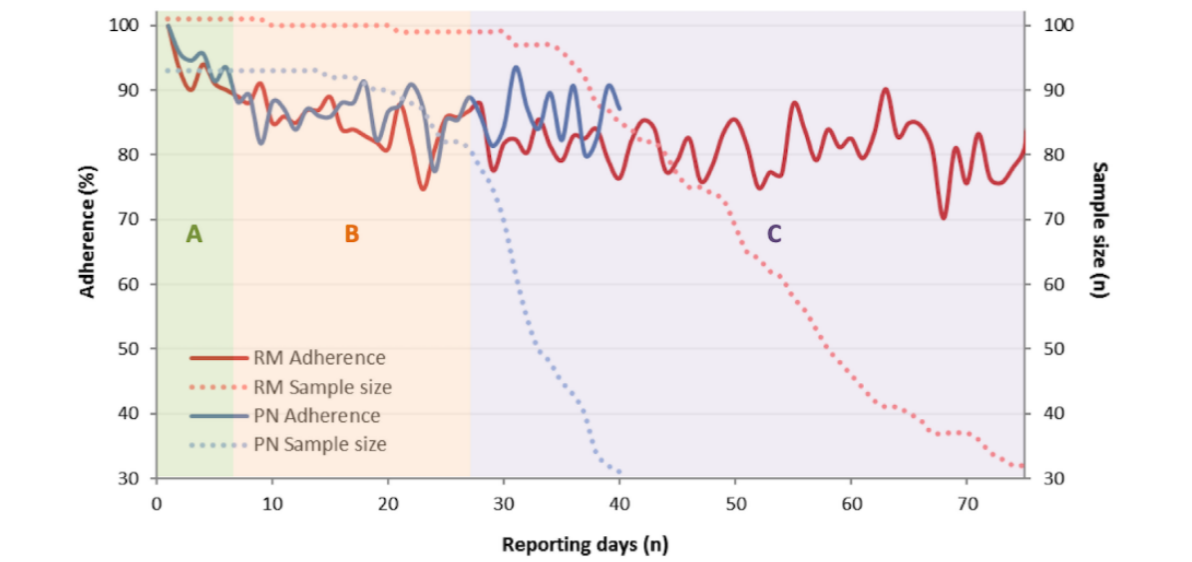
Adherence (%) by reporting day and study center. It is possible to describe three phases (indicated by light background color): the first phase (A), lasting 6 days, during which adherence fell from 100% to 90%; the second phase (B), lasting approximately 20 days, during which adherence fluctuated until reaching 88%; and the final phase (C), during which it slowly declined to 80%. RM: Rome; PN, Pordenone.

The mean prescription period was longer in Rome than in Pordenone (76.2, 95% CI 70.4-82.0 vs 53.9, 95% CI 50.1-57.7 days, respectively). The pattern was similar for the mean reporting period (Rome: 70.6, 95% CI 64.9-74.4 vs Pordenone: 48.2, 95% CI 44.6-51.7 days) (Figure 2). Mean adherence levels were 85.7% (SD 13.9) in Pordenone and 82.3% (SD 13.7) in Rome. The analysis of mean adherence values by reporting day showed a similar trend for both participating study centers. In Rome, the adherence trend by reporting day displayed 3 different phases: phase A, a first phase of 6 days with an adherence 93.1% (564/606); phase B, a second phase of approximately 40 days, during which the adherence fluctuated around 83.65% (2834/3388); and phase C, a final phase of slowly declining adherence, oscillating around 78.55% (1952/2485). Pordenone’s adherence trend by reporting day followed the same evolution for phases A and B. Due to a shorter pollen season and mean prescribed period, we did not investigate phase C in Pordenone (Figure 2).

**Figure 3:**
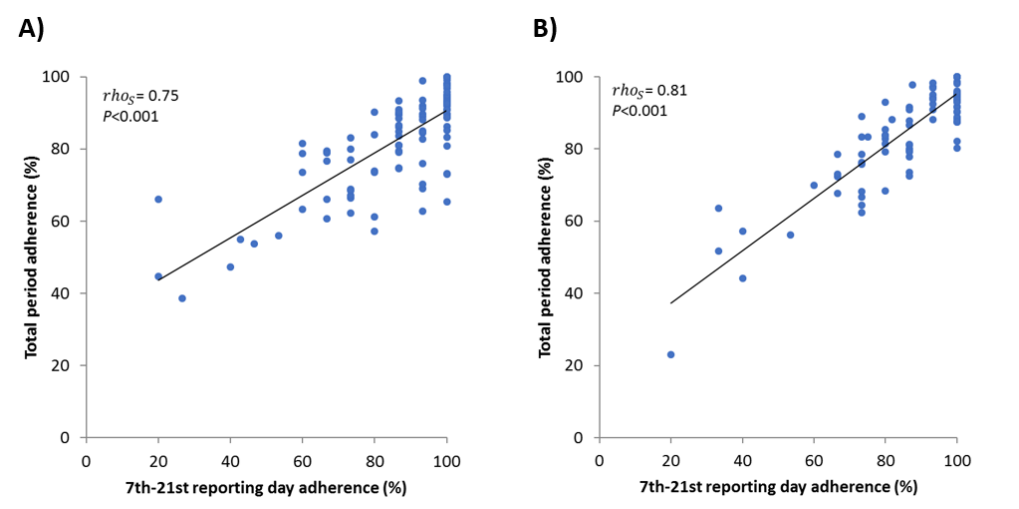
Correlation between adherence achieved between the seventh and the 21st reporting days and total reporting period adherence, by study center: (A) Rome (n=101); (B) Pordenone (n=93).

**Figure 4:**
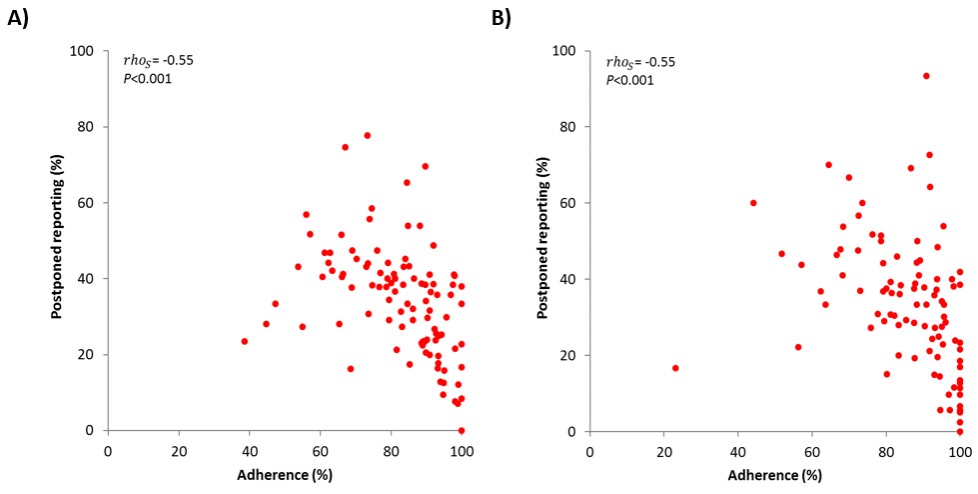
Correlation between postponed reporting (%) and total reporting period adherence (%) by study center: (A) Rome (n=101); (B) Pordenone (n=93).

**Figure 5:**
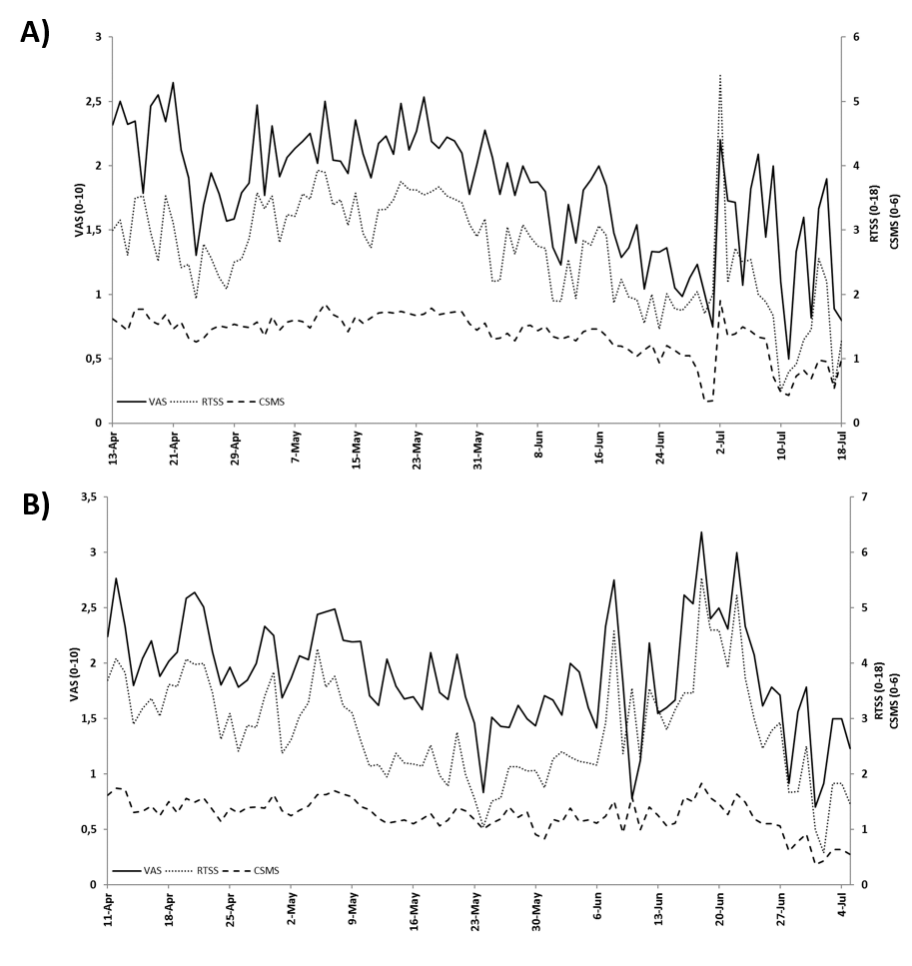
Mean visual analog scale (VAS) score, Rhinoconjunctivitis Total Symptom Score (RTSS), and combined symptom and medication score (CSMS) by time considering the local whole season of grass pollen in (A) Rome (n=101) and (B) Pordenone (n=93; see Figure 2 and Table 2 for definitions).

Interestingly, the total adherence was directly proportional to the adherence assessed between the seventh and 21st reporting days (Spearman ρ=0.75; *P*<.001 and ρ=0.81; *P*<.001 for Rome and Pordenone, respectively) (Figure 3) and inversely related, although with less intensity, to postponed reporting (ρ=–0.55; *P*<.001 for both Rome and Pordenone) (Figure 4). In both populations, the mean RTSS, evaluating symptoms of the eyes and nose, showed a parallel trend with the mean VAS scores assessing the general disease-related impairment. Also, the CSMS followed a similar trend but with less distinct variance (Figure 5).

**Figure 6:**
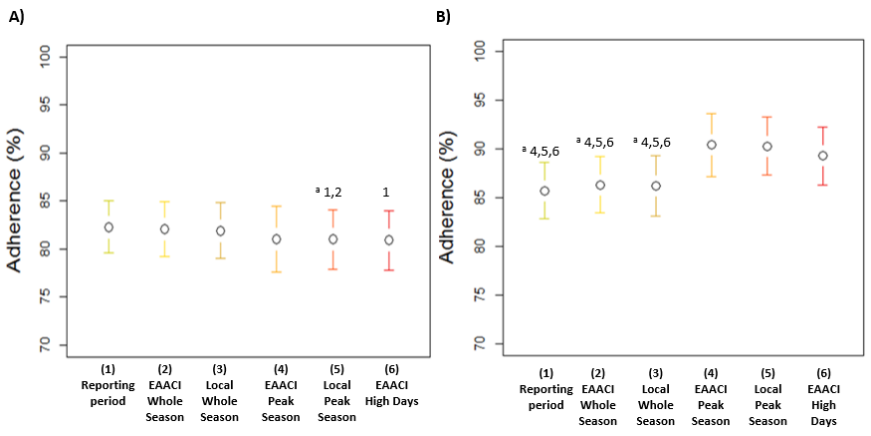
Mean (95% CI) adherence to recording of the e-diary during the pollen season in children affected by seasonal allergic rhinitis in (A) Rome and (B) Pordenone. Adherence was calculated for each patient considering the total reporting period and according to whole, peak, and high days of pollen periods defined by the European Academy of Allergy and Clinical Immunology (EAACI) and local criteria. See Figure 1 and Table 2 for criteria. "a" indicates that nonparametric Friedman test for repeated measures was applied and only statistically significant *P* values of multiple comparisons, adjusted by Bonferroni correction, are highlighted.

Mean adherence values differed only slightly in Rome during the different pollen periods (Figure 6, part A). By contrast, adherence values were significantly higher in Pordenone during the peak pollen season and the high day (Figure 6, part B).

## Discussion

### Principal Findings

In this bicenter study, we investigated the adherence of Italian patients with SAR to symptom and medication monitoring via an e-diary prescribed by their doctor in the context of an observational study. We found that adherence to recording was (1) very high (>80%) in the first 7 weeks of monitoring, (2) predicted by the adherence in the first 3 weeks of the monitoring period, (3) inversely associated with the frequency of delayed e-diary compilation, and (4) higher during the peak pollen season.

The trajectories of the mean adherence to recording were highly similar in both study populations, notwithstanding their differences in geographical location (northern vs central Italy) and age (adults vs children). Moreover, we observed only a few patients with very low adherence to e-diary recording, that is, failing to register their symptoms during more than 60% of days within their monitoring period (not shown).

This level of adherence is at great variance from levels published in previous studies on e-diaries in patients with allergic rhinitis who had not been specifically instructed and advised by a doctor to use an app. With this approach, the Mobile Airways Sentinel Network observational pilot study among 2871 allergic users from 15 countries reported an adherence to symptom recording of only 9.5% after 14 days of recording [14]. A follow-up project among 9122 users from 22 countries showed that only 16.4% of the users were still recording their symptoms after 14 days [15].

Digital technologies have been shown to be a very useful tool for the assessment of real-life data among big patient groups [14-17]. While the patient-initiated use of an e-diary app may be very helpful in highly motivated patients looking for self-management opportunities, it seems that this scenario applies to only a minority of the users spontaneously downloading, installing, and using an e-diary app for allergic rhinitis [14,15]. However, our results showed that in a blended care approach combining face-to-face visits with internet-based support technologies, patients are keen and able to correctly use an e-diary when contacted and instructed to do so by their allergist. It has to be underlined, though, that our patients were participating in an observational clinical study and we do not know whether their high adherence would have been also maintained in the context of routine clinical practice. This hypothesis deserves to be tested in a real-life or surveillance study.

The adherence to e-diary recording of the patients in Rome was slightly, but significantly, higher during the grass pollen peak season, when allergic symptoms were also more severe. This observation may be easily explained by increased awareness and motivation linked to symptom severity. This outcome should be taken into account when considering the use of e-diaries outside the pollen season or in patients with very mild symptoms. With regard to monitoring scores, we demonstrated that the overall VAS score reliably reflected the results of the RTSS and CSMS, which confirms the usefulness of VASs for digital symptom assessment as previously shown in other studies [31,32].

Of great relevance is, in our opinion, that the overall adherence of a patient to e-diary recording over a period of 2 or more months can already be predicted with enough confidence in the second and third weeks of monitoring. Patients at risk of poor adherence could therefore be identified and receive supplementary information and education, thus facilitating a higher compliance.

### Limitations

First, our study population consisted of Italian patients only, so that our results now require further evaluation in different cultural contexts. To this end, we are examining the outcomes of a similar study performed in 7 southern European and Mediterranean countries. Second, we cannot comment on possible outcome improvements, as the study did not include any control group. Third, we limited our monitoring period to a maximum of 90 days; we do not know whether the patients’ adherence to recording would have remained high enough beyond this time frame. Fourth, our results and proposal cannot be applied to SAR patients not using a mobile phone. Fifth, we did not evaluate potential adverse effects of the use of an e-diary, such as excessive attention to disease or even facilitation of anxiety and obsessive disturbances.

### Conclusion

Our study showed that adherence to the daily symptom and medication monitoring via an e-diary was maintained at a high level up to 2 months by SAR patients properly informed and educated by their allergist. This outcome underlines the strength of a blended care approach and needs now to be confirmed in a real-life clinical allergy setting. Our results contribute to reinforce positive expectations for a proper use of mHealth technology in monitoring patients with SAR for diagnostic and therapeutic purposes.

## Abbreviations

ARIA: Allergic Rhinitis and its Impact on Asthma
CSMS: combined symptom and medication score
EAACI: European Academy of Allergy and Clinical Immunology
RTSS: Rhinoconjunctivitis Total Symptoms Score
SAR: seasonal allergic rhinoconjunctivitis
VAS: visual analog scale
